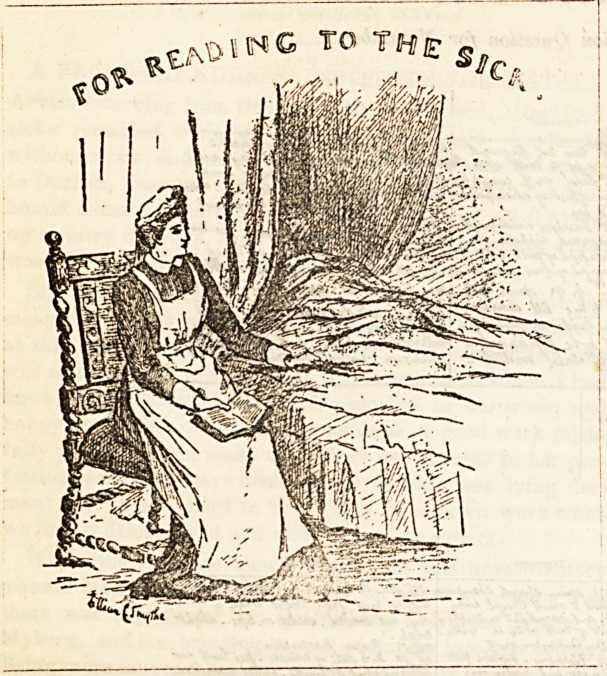# The Hospital Nursing Supplement

**Published:** 1892-01-02

**Authors:** 


### "The Hospital" Nursing Mirror.

Being the Extra Nursing Supplement of "The Hospital" Newspaper.

Contributions for this Supplement should be addressed to the Editor, The Hospital, 140, Strand, London, W.C., and should have the word "Nursing" plainly written in left hand top corner of the envelope.

### En Passant.

NURSES' HOLIDAYS.—The completion of the new buildings at the London Hospital adds twelve more bed-rooms to the accommodation provided for the nurses, and the nursing staff has been recently increased by that number. This fact enables the Committee to carry out their long cherished desire of granting three weeks' annual holiday to all their staff nurses.

KEEPING CHRISTMAS.—The Students' Club of Charing Cross Hospital gave an entertainment to the patients and nurses just before Christmas. The farce, " Turn Him Out," and the comedy " Our Wife," were excellently acted and repeated on three successive nights, so that all should have a chance of seeing it. Sister Catherine and others sang in the intervals, and very happy evenings were spent. The pretty uniforms of the nurses and Sisters were much admired by the visitors. At the Royal Free Hospital, the students (who are women) gave an entertainment on December 17th, which was much enjoyed, Mother Hubbard and her dog, and other old favourites receiving a hearty welcome.

SHORT ITEMS.—Sister Frost and Miss Holditch desire to thank all those who have sent them Christmas greetings.—Almondbury has started a district nurse ; Nurse Longbourne, of the Huddersfield Nurses' Home, has been chosen for the post.—A sale of work has been held in aid of the Kingston Nursing Association ; it was well managed, and the net takings were £44.—This month Miss Minet concludes 20 years cf good work in connection with the Stratford Nursing Home and Children's Hospital ; the occasion is to be marked by the presentation of a testimonial.—Dr. Broadbent will take the chair at the first annual meeting of the Nurses' Co-operation to be held at 20, Hanover Square, on January 19th, at four p.m.—We desire to acknowledge 10s. for the Nurses' Bed from Miss Bishopp.—The first annual meeting of the Campbeltown Nursing Association has been held ; 56 patients have been attended by Nurse Mackay.—It is proposed to turn the board-room of the Ipswich Hospital into a nurses' dormitory, instead of building a proper nurses' home. —Dr. Goodall has been elected Dr. Collie's successor at the Eastern Hospital.—The Gloucester Charity Trustees have decided to have a Matron, instead of a Master, for the United Hospitals.

NURSES IN MASHONALAND.—In last Monday's *Times* was a long letter from Miss Sleeman, dated August 11th, from Umtali Hospital, Manicaland. She and Miss Blennerhassett and Miss Webley had walked the last 190 miles of their journey, only to find at the end of it they were not wanted so far as nursing went. We quote the conclusion of the letter : " Words cannot describe how good all are to us, but I am afraid we must make up our minds to a hard time during the rains, for food is getting short even now, and the wagons do not seem to have any idea of being here before then ; the roads are impassable from November to March. Unless they get an enormous supply of food we shall have to depend on Kaffir meal, a little rice, and pumpkins ! Our fly-bitten oxen have come to an end, and it used to be so difficult to cook it, for we have no oil, lard, butter, or fat. Lately we have lived on 'bully beef !' At present there is nothing for us to do in the way of nursing, and I am afraid we shall have very little to do this winter, as people are afraid to come up because of the starvation. Of course, we are very glad that there is so little illness, but I think that we have come up a year too soon ; however, here we are, and this last experience has been terrible. . . . No chance of seeing our boxes for months, perhaps even never. The men think it terrible for themselves, and so pity us tremendously. . . . During the rainy four months we shall be shut in like a beseiged city, and live on mealie meal porr idge made with water—and great joy if we can get a pumpkin. . . . I am afraid this letter will seem rather like grumbling. Only the mere fact of writing home is almost more than we can bear. We try to be cheerful and hide our miserableness, for every one is so nice to us. . . . They make so much of our walk, that we begin to wonder if we have really done anything very wonderful. We sleep in two tiny mud rooms. Our beds are made of six wooden posts stuck into the ground, and short pieces of cane tied across the top, then a Kaffir mat made of split bamboos for a mattress, and there we are. It is a great improvement on the ground, at any rate ! "

" THE YEAR THAT'S AWA'."—The old year has seen many lives laid down in the nursing cause ; on its first day a young probationer of twenty died of typhoid contracted on duty, and last week we chronicled the death of a young Glasgow nurse. Nurse McArthur and Miss Orr died of typhus caught in following their calling ; Nurse Chorlton died of scarlet fever. Sister Catherine, of Devonport, and Sister Roberts, of Bradford, were victims of typhoid. Miss Derham, who founded the Convalescent Home at Bushey ; Miss Prince, for many years Matron at Blackburn : Miss Heanley, to whom the Boston Hospital owes so much—the old year has taken them all from us, and many more besides. No need to linger over the roll-call ; they went willingly ; every nurse knows that she must give her life if her work demand it, and long is the list of the unknown martyrs who have quietly won the crown. The old year has deprived us of the labour of some we could ill spare, but it has been a year of steady progress, and the nursing ranks are now more firmly knit, its members loyally bound together to take a high and noble view of their calling. We can look back on another pleasant gathering at Marlborough House, on the steady success of the Nurses' Co-operation, on the rapid advance of the asylum attendant. Indian nurses have received special recognition ; Lord Tennyson deigned to help the nurses in their present to Princess Christian's daughter ; district nursing in villages has been forwarded by the formation of a rural branch of the Queen Victoria Jubilee Institute. There has been some grumbling in the nursing ranks, but the general tone has been loyal and true, and all authorities seem anxious to do what they can to promote the welfare of their nurses. In conclusion, we would quote some verses by Rudyard Kipling ; if a novelist can thus regard his work why not a nurse ?

One stone the more swings to its place
In that dread Temple of Thy worth ;
It is enough that through Thy grace
I saw naught common on Thy earth.

If there be good in that I wrought,
Thy hand compelled it, Master, Thine ;
Where I have failed to meet Thy thought,
I know, through Thee, the blame is mine.

One instant's toil to Thee denied,
Stands all eternity's offence,
Of that I did with Thee to guide
To Thee, through Thee, be excellence.

### A Nurse in Natal.

*(Concluded from page lxxvi.)*

### A PROPOSED NURSES' INSTITUTE FOR NATAL.

AFTER returning from the case I last mentioned, Miss Goodricke remained with her aunt in their single apartment, without work, and uncertain what to do. She could not go to Durban, because she had been warned against the hot, humid climate so injurious to new comers; and all the cold, up-country districts, even if they offered any opening, which was very doubtful, were likewise closed.

She could not return to England, for she had not the means. A great change was perceptible in Miss Goodricke at this time. Disappointed, dejected, and unoccupied, she was scarcely to be recognised as the same woman whom I had known in this country—hopeful, absorbed in her work, and happy as I think only the consciousness of good work faithfully performed can make us. Even her interest in her profession seemed to have declined, or at least was lying dormant; for it is so hard to keep faith in our own work when we find it discouraged and undervalued by others.

While the days are slowly passing in this unsatisfactory manner Miss Goodricke was greatly surprised at hearing that there was a proposal for establishing a Nurses' Home in Myberg, and for bringing a staff of nurses into the colony. Bring more nurses when one seemed unable to make a living there—what a strange scheme this appeared!

Yet, on the other hand, how great a boon such an institution should be to the country, if only the people themselves could appreciate, and heartily support it! This plan, originating with some of the highest and most influential persons in the colony, naturally gave rise to much discussion; opinions being, of course, of a very varied character. Some of these I one day heard exchanged between a group of ladies and gentlemen who, like myself, were met in a public building in the town.

"What do you think of this idea of bringing out trained nurses to the Colony?" asked one. "Are you going to subscribe?"

"Not I," said a hard-featured man, "I want no trained nurses. When I am ill, I have got my wife or my daughter to nurse me, and want nobody else."

"I do not quite understand the subject," said a quiet looking lady. "How is it proposed to manage the matter? It will cost a great deal of money."

"A considerable sum of course will be wanted at first, for erecting a building and getting the nurses here; but we shall have no difficulty in raising that."

"But when the building is finished, and the nurses are here, what is to keep the institution going? That is the part I do not understand," said the quiet lady, who, notwithstanding her evident timidity, seemed to like to get to the bottom of things.

"Oh, nothing can be simpler," said the sanguine gentleman, whom I easily saw to be one of those who wish to look only through their own spectacles. "The institution, of course, will be self-supporting, after it is once started. People will send for a nurse when they want one, and we shall have a fixed charge, say £2 2s. per week (that is the usual thing I believe), and, with the addition of a few voluntary contributions, we shall do very well. Of course the people will be only too glad to have the nurses when they can get hem."

"I am sorry that I cannot quite agree with you," said an earnest voice, and looking round, I now for the first time perceived the presence of one of the leading medical men of Myberg. "I am not so sure that people will want them."

"Of course they will, Dr. Easton, there can be no doubt of that," said the former speaker, who was very tall and thin. "Why, you cannot be ignorant of the many distressing cases that have occurred during this summer, when an efficient nurse would have been the greatest possible boon."

"I know all about that," said Dr. Easton, "but I also know that when there have been trained nurses in the town they have been almost totally unemployed. How do you account for that? It is not because they were unknown, as some would say, for we all knew of their being here, and have recommended them to our patients. But the people do not want nurses."

"It is very strange, very strange indeed," returned the thin gentleman. "There must have been something wrong about themselves; either they were not good nurses or there was something about them which people personally disliked."

"You scarcely seem to approve of the scheme, Dr. Easton," observed a second lady. "Do you dislike trained nurses for your cases? I have heard that some doctors do."

"I see you do not quite understand my views," replied he. "As for disliking trained nurses, I do not see how any doctor can do that. We know too well how much of our own success depends upon the nursing. We can give orders, but we cannot carry them out. It may not perhaps matter to Dr. Matthews (who, by the way, really is against this scheme) because if he has a bad case he has only to say, 'go to the hospital.' But we cannot do that. We have to put up with what we can get, which is perhaps a small room in a boarding-house, and a woman, calling herself a nurse, who will probably let us find on our next visit that, instead of the milk diet we have ordered for our fever patient, a lump of steak or a mutton chop has been given! Such ignorance of and disobedience to orders would be impossible in any trained nurse worthy of the name. With regard to the present proposal, I should think the plan a most admirable one, if only I could hope that it would be successfully carried out."

After this animated speech of the doctor's, no one seemed to have any further remark to make, and the whole party presently moved away. I could not but think myself that Dr. Easton's words contained the pith of the whole matter.

To make a Nurses' Home successful, it is obvious that the community at large, not a few large-hearted and benevolent persons, or a scattered family here and there, must strive to estimate at its real value the great blessing which it offers to society; and resolve to support it in a philanthropic and generous spirit.

I was sorry that our domestic affairs rendered our departure from Natal necessary shortly after this, so that I could not personally watch the progress of a movement in which I felt considerable interest. As we left the shores of that sunny little land, which, with all its faults and shortcomings, one feels compelled, after even a short residence there, to love, I could not but hope that before the scheme is accomplished, Natalians will awake from their coldness and apathy, to value and support, as other peoples do, the new advance in civilization.

I shall then regret that one for whom I could not help feeling some sympathy, will not be there to share in that brighter and more hopeful era.

When I last heard from Miss Goodricke, despairing of gaining a livelihood in Natal, she had obtained—through the interest of an old Johannesburgh patient—an engagement on the nursing staff in Mashonaland: whether she will ever return from there is doubtful.

### Leaves from a Nurse's Case-Book.

*(Sent in answer to the Examination Question for November.)*

### THE NEW YEAR.

THE old year has gone! The New Year has come! What varied thoughts rise in our minds at these words! The 365 days just ended, which opened perhaps with glee and rejoicing, have closed in sorrow, and we are moaning over our lost health, lost hopes, lost possibilities; all those things we counted on and valued so much are gone. Let them go, but remember, dear friends, we are beginning a new term of life which, though commencing in grief and suffering, for we may not have quite passed through the night of doubt and sorrow, yet

"Clear before us through the darkness,
Gleams and burns the guiding Light."
"Joy cometh in the morning."

Cheer up, then, and strive to make a better use of our opportunities in the future, for a good deal depends on ourselves. "Man is man, and master of his fate," says the poet. There is no blind fate driving us on whither we know not, but a living, all-seeing God watching over us, a God who created for His good pleasure, intelligent beings with the power to choose betwixt good and evil. The heathen had a notion of the truth when they made the fable of the man, whose cart-wheel having come off, called loudly on Mercury to come and help him out of the rut. The god appeared but upbraided him for lying still and doing nothing, when if he had put his own shoulder to the wheel he would soon have been out of the mire.

In the coming year let us put our shoulders to the wheel, and do our part not to fall into sloth and discontent, for it is so easy to depend on others, and say "do help me," much easier than to make up our minds to help ourselves. We must make a high resolve, and then ask our heavenly Father to give us power to keep these good resolutions. This also is necessary, for except the Lord build the house their labour is but lost that build it, except the Lord keep the city the watchman waketh but in vain. We shall, indeed, find it lost labour if we only act in our own strength; we must *will* and then we can do all things through Christ, who strengtheneth us.

Our resolve then for New Year of 1892 shall be to gain a patient, contented heart, cheerful and grateful with love to God and man for the mercies and benefits received from Him through His creatures, and our prayer as follows:

In our weakness and distress,
Rock of strength be Thou our stay,

Keep us faithful, keep us pure,
Keep us evermore Thine own,
Help, O help us to endure,
Fit us for Thy promised crown.

### Distributing Christmas Parcels.

WHAT a terrible fog it was! It got into your eyes and made them smart, and it got into your throat and choked you, and its simply ruined the looks of the shops, and kept all the would-be shoppers at home. But we didn't care: the long office table was piled with Christmas parcels, and we knew that in spite of the weather we were going to make such lots of people happy! Parcels from E.L., J. A. Luton, (cushions made by two little girls of six), Miss Little, and C. G. had been added on the 23rd, and on the 24th arrived three boxes from Madame Monchablon, one box contained eleven lovely dolls of all sizes and most beautifully dressed, and we promptly forwarded them to the North-Eastern Hospital for Children; a second box contained warm shirts, and shawls, and stockings, and socks, and all sorts of warm clothing; this we sent to the Poplar Hospital; the third box contained some scrap books, which we took to King's College Hospital, and some bedside mats, which we fancy will be most useful to district nurses. There were also some type-written linen books by Madame Monchablon's staff, and we send our hearty thanks for the very handsome addition to our pile of parcels. The parcels fairly filled a four-wheeler, and we set off on our travels quite confident that if we did come to grief in the fog the parcels would protect us from injury. First to King's College, where the Sister-Matron was loud in her thanks to our readers; then on to the Middlesex where we left a miscellaneous parcel in the Secretary's office, and a special bundle in the Queen wards. When we left the Middlesex, the cabman tentatively suggested St. Mary's. St. Mary's! why we had quite forgotten it! "It won't take me long to drive there, and its a nice 'orspital; I was in Lewis Lloyd ward once." And so off we went to Paddington and left a special bundle for the Lewis Lloyd ward. Back to Charing Cross where the Matron was discovered in the chapel busy talking over decorations; here we left the prize shirt and the prize dressing-gown, besides lots of lovely socks. Then on to Westminster, and as we mounted the stairs to the Matron's office, we heard the sweet sounds of the Christmas hymns floating down. There was evidently a choir practice going on. The fog by this time was almost impenetrable, so dismissing the cabman who had been so faithful to St. Mary's, we went East by the Underground. At the London Hospital there was a parcel for Charlotte wards, and a bundle of socks for the George wards. Everywhere were signs of the coming festivities, and house-physicians, house-surgeons, Sisters, nurses and patients, were alike engaged in making evergreen wreaths. To Victor ward we took Nurse Lee's dressing-gown and a few warm petticoats, and very, very heartily did Sister Victor appreciate the parcel. "Nurses know so well what we need," she said. There was a sort of hush over Victor ward, for an operation of a serious nature had just taken place. Nurse Ayrton's garments for children went down to Shadwell, a parcel of socks and shirts and comforters for the Seamen's Hospital, a mixed bundle for the Metropolitan, and a small parcel for the little hospital at Hounslow. Altogether we had 250 articles to distribute this year, as against 15 last year. We heartily thank all our readers for their co-operation, and assure them that everywhere their gifts were warmly appreciated. When in the midst of the darkness of the London fog we opened parcels from Coldstream in Scotland, or from Tunbridge Wells, we thought of how lovely the country must be with the bright sunshine on the frosted trees and the air exquisitely keen and clear, and then we thought that those who enjoyed those advantages of nature might well do something for those sick in fog-choked London. Applications for parcels came from Norwood and Windsor, two rich neighbourhoods without the metropolitan area, so we thought ourselves justified in refusing them, and confining the gifts to dwellers in the great city. We should like to linger over the description of the neat knitting of Miss A. F. Heanley, the useful garments made by Nurse Elizabeth Bishop, the socks knitted laboriously by childish fingers; the kindness which prompted some of our contributors to fasten a Christmas card to each garment. But space forbids; yet be sure that no stitch or thoughtful shaping of any of this clothing is lost; those to whom the garments have gone have not so many presents that they are likely to leave unnoticed the perfectness of these gifts from our readers.

### At Duty's Call.

[Written to commemorate an incident which occurred at Charing Cross Hospital during an entertainment given by the Students' Club on Wednesday evening, December 16th, 1891.]

HARD by the spot where stood a cross,
Which good King Edward gave
In memory of his dear wife
Then passing to her grave,
A noble building stands to-day—
The cross its emblem still—
Within whose walls the indigent
Find balm for every ill.

For there kind hearts and skilful hands
Turn sickness into health,
And there the simple poor may find
What's better far than wealth.
Within its friendly portal I
Of late did speed, a guest,
To join a throng that gathered at
The Students' Club's request.

The board-room was both bright and gay,
And fitted with a stage,
To cheer the sick with echoes from
The Comic Muse's page.
Upon the stage a gallant stood,
A nobleman of France,
Who told a maiden fair how love
Consumed him in her glance.

The maiden coyly answered him
That she would only wed
With one who rich and handsome was,
And nobly born and bred.
"Alack!" the gallant cried, "am I
Not rich and noble too?
And handsome?—Well, of course, sweet girl,
That must be left to you."

Out spake the gallant's noble friend—
"Mon cher ami," quoth he,
"If this fair lady you *must* wed,
Just leave it all to me.
She shall be yours, I promise you,
If you will but retire
For fifteen minutes, thus you may
Obtain your heart's desire."

The gallant laughed, "ha! ha!" cried he,

"Now can't you make it *ten?*

For fifteen's long enough to try

The patience of most men!"

And thus this jovial play went on

So full of fun and glee;

The audience sat and shook their sides,

They laughed so merrily.

But lo! a change crept o'er the scene,

A call to duty came;

In haste a messenger arrived,

Upon his tongue a name.

From lip to lip, from man to man,

That name—'twas Johnson—passed;

An "accident" had just come in,

And aid was wanted fast!

But there they found him not; and then
A sweet-faced nurse arose,
A noble woman fitly trained
To solace human woes.
Right cheerfully she left the throng
And chose the nobler part
Of gentle ministration, dear
To each true woman's heart.

Meanwhile the play had sped apace,
The plot had thicker grown;
The maid was now a marchioness
A coronet her own.
But hark! once more the summons came,
A whisper was passed round,
Another gentle nurse stood up,
Obedient to the sound.

And thus they played, and thus they toiled,
Until the night was spent:
A duty here, a pleasure there,
And joy with sorrow blent.
A scene within a scene is life,
A wheel within a wheel!
While some rejoice, some weep and mourn,
Such misery they feel!

ALBYN MULLOY.

### Everybody's Opinion.

*[Correspondence on all subjects is invited, but we cannot in any way be responsible for the opinions expressed by our correspondents. No communications can be entertained if the name and address of the correspondent is not given, or unless one side of the paper only be written on.]*

### THE PRINCESS'S LETTER.

"NURSE F." writes: Being one of the Second Thousand I wish to send my thanks for the autograph letter of H.R.H. our President given with this week's HOSPITAL. I think it very good of you to publish it in this way, so that we all get a copy. Surely the graciousness of our President is unparalleled.

### TRAINING FOR NURSES.

"L." writes: Many, besides myself, will be glad to be assured that the Nurses of the Salvation Army receive the training that is at least generally considered necessary, viz., one year. May I ask if the same can be said of the Church Army Nurses, in whom some of us have a still greater interest? At one time, six weeks' experience (for it could not be called training) was gained in a poor-law Infirmary, consisting almost entirely in bed and poultice making, and then they were called, and went out as nurses! Some of us at the time the plan was formed ventured to expostulate with the authorities as to the dangers of such so-called "training," and I trust that experience may have modified these plans also; but we should be very glad to be assured that it is so. The earnest, and I believe well-founded, opinion of many is, that nursing and mission work are duties, or processions, that are best kept apart and exercised independently.

### Appointments.

[It is requested that successful candidates will send a copy of their applications and testimonials, with date of election, to THE EDITOR, The Lodge, Porchester Square, W.]

DUNEDIN HOSPITAL.—Miss Edith Mawe has been appointed Matron of this hospital of 120 beds in New Zealand. Miss Mawe was lately in charge of the surgical division of the Colonial Hospital at Gibraltar; she is an excellent nurse, a pleasant fellow-worker, and an intelligent companion. We congratulate Dunedin on its choice.

MISS D. HAMILTON RAMSAY has been appointed staff nurse in the Edinburgh Royal Infirmary.

### Told on New Year's Eve.

"Tired, Sister?"

"No—at least a little—but—"

"But what?"

"Thinking."

"Of what?" I asked, regretting almost immediately the question, as a look of even deeper sadness crossed Sister St. Monica's face; also as I remembered what night it was—the last of the old year—a day generally sacred to reminiscences of the past as well as for hopes of the future.

"Why, of many things," was the answer given, lightly enough. "One—that we have talked too long, and there is work to be done," and so in a few minutes we had left the little sitting-room off the ward, where we had been having some tea, and went to our evening's work.

The wards were bright with Christmas decorations, but to my mind none came up to St. Monica, and there was a general air of rejoicing through them all. Yes; even in the midst of so much suffering, one really cannot realise the brightness of the Christmas festival in a hospital, unless there. Why, even old men and women looked forward to, and enjoyed it as eagerly as the inmates of the children's ward, and I often wondered why outsiders do not think more about it, and send their offerings to cheer the suffering inmates, as much by the kind thought as anything else, for although, as Keble says, "the world's a room of sickness," it need not be too gloomy a one. Certainly the atmosphere of the festive season was still present, and on the next day there was to be a tea and entertainment in the Board-room to those who could join in it, and I need not say those who could not would not be forgotten.

"We shall not sleep to-night, Nurse," said one patient, "at least not till after twelve, and I shall be the first to wish our night nurse a happy New Year."

"But supposing she ain't in the ward?" said another—the inmate of the next bed.

"Oh yes, she will be," answered the first speaker, "I shall call her a little before twelve, and say I am took awful bad."

And so the day's work ended, and saying a general good night—"until next year" called out my youngest and pet patient—I left the ward, and went as usual to Sister's room to say good night also. She kept me talking a little, saying as I left,

"May your New Year be happy, little one—only bright days for the future—with no sad 'looking backs.'"

"Little one!" and I twenty-seven! "No looking backs"—if she only knew!

I somehow always felt sorry for Sister St. Monica; she never seemed to find the happiness in her work she ought to have done, *well* as she performed her duties. It always seemed as if she were burying some grief in it which had given her, at least outwardly, a hard manner; but I was very fond of her, and for the three months I had been in her wards she had been very good to me.

In the dormitories there was life, most of the nurses having begged to have been excused supper, only to have a private one upstairs, and were as so many school-girls. I would not join—I was tired; besides, I wanted to "think my little think" on this the last night of the year. Opening my window, I looked out over the city lying white in the snowy moonlight. Already the bells were beginning—the passing bell of the dying year—ringing their knell of hopes departed to so many, and to as many bringing fresh hopes, that the coming year would bring all they wanted.

I knew what I wanted—what I have wanted for the last five years—an ever present want—yet more vivid on this particular night, and as I gazed out it seemed as if it must come soon—I must know—or else—and yet I was very content with my life of the last four years.

Soon the dormitories were silent, "for when people have work to do they cannot afford to be sentimental," said bright little Nurse Harding, when asked if she would wait up to see the New Year in; but still I stayed on, though over the city the hour of eleven had sounded. "It will not matter another hour," I thought—"only an hour—and yet—"

A hushed step in the corridor, a subdued knock at my door, and the night Sister entered, saying,

"Not in bed, Nurse Yorke; that is well, for you are wanted."

"For duty?" I asked, laying my hand on my cap, which I had taken off.

"No," she answered, "you are wanted in St. John's Ward. A patient arrived this evening, and is evidently dying, and has asked for you."

(*To be continued.*)

### Examination Questions.

We have not received a single set of correct answers to our riddles, so are obliged to answer them ourselves: (1) What drug is remarkable for lengthening the life of scholars? Belladonna, because it causes the pupil to dilate. (2) What is the difference between cantharides and a lottery ticket? The first draws something, the second nothing. (3) What two opposite evils are caused by match-making? Phosphonecrosis or decrease of jaw, and curtain lectures or increase of jaw. (4) Of what bird does paracentesis remind you? The woodpecker tapping. (5) Why does orthopnœa cause honesty? Because it causes the patient to prefer to be upright. (6) Why is pyrosis like a love affair? Because it is marked by heart-burn. And now to depart from frivolity and set a serious question for the good of probationers:—"Mention the early symptoms of smallpox, scarlet fever, and measles." Answers must reach this office by January 23rd; the best answer will be printed and a book sent to the writer.

### Presentations.

The Lady Superintendents (Mrs. L. Smith and Miss Duncan) of the Blackheath and Richmond Private Nursing Institutions have been presented by a number of their nurses with an exceedingly handsome set of fish knives and forks. They are, naturally, very much gratified, not only by the beauty of the gift, but by the kind feeling of which it is the manifestation.

On Christmas morning the pupil midwives and nurses of Queen Charlotte's Hospital presented the Lady Superintendent (Mrs. Phillips) with a pair of handsome Dresden china candlesticks and silver cabinet photo frame. Mrs. Phillips was very much pleased, not only with the presents, but also with the kind feelings and good wishes of her nurses. She thanked them in a very impressive manner.

### Amusements and Relaxation.

| Names. | Dec. 24th. | Totals. | Names. | Dec. 24th. | Totals. |
|---|---|---|---|---|---|
| Lightowlers | 15 | 538 | Jenny Wren | 16 | 460 |
| Bonne | 16 | 539 | Darlington | 15 | 534 |
| Morico | 15 | 607 | Nurse G. P. | — | 99 |
| Robes | — | 143 | Hetty | 16 | 451 |
| Dulcamara | 15 | 551 | Janet | — | — |
| Psyche | — | 7 | Jackanapes | — | 367 |
| Agamemnon | 15 | 577 | Ex-Nurse | — | 99 |
| Nurse J. S. | 14 | 513 | | | |

### Notice to Correspondents.

Results of the fourth and last quarterly word competition will be published in our next issue.

## Figures and Tables

**Figure f1:**
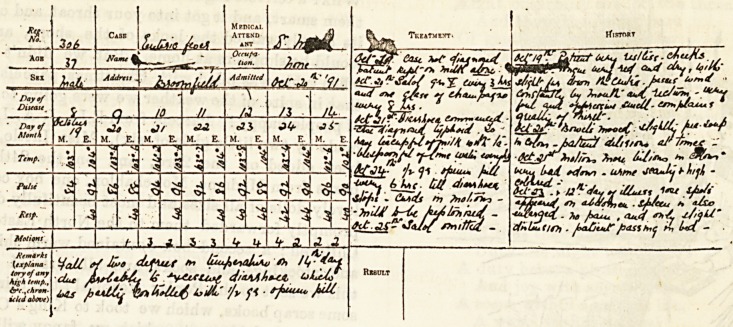


**Figure f2:**
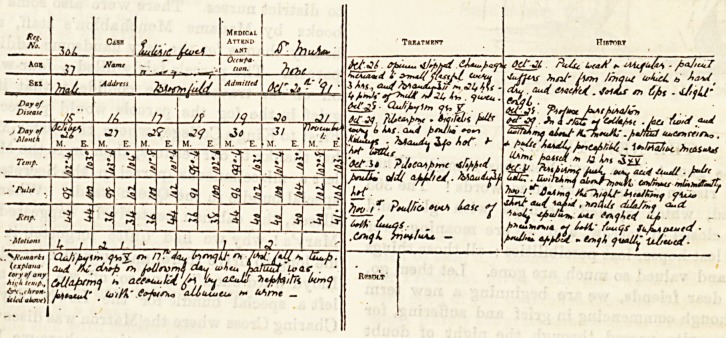


**Figure f3:**
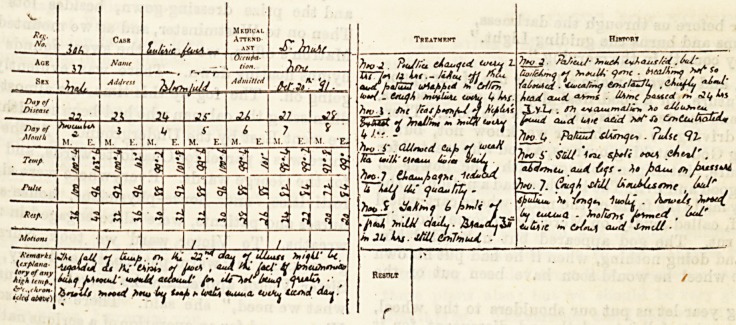


**Figure f4:**
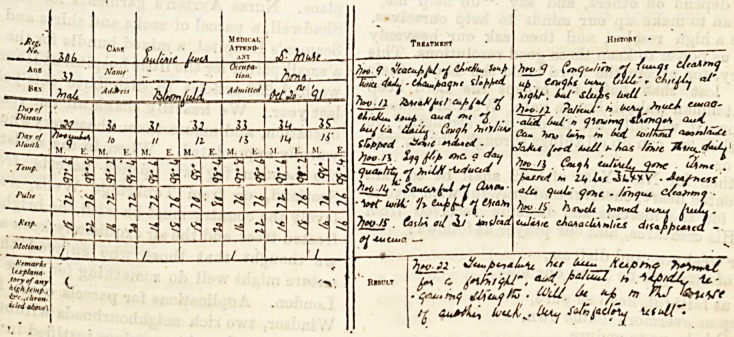


**Figure f5:**